# A pooled analysis of 10 case–control studies of melanoma and oral contraceptive use

**DOI:** 10.1038/sj.bjc.6600196

**Published:** 2002-04-08

**Authors:** M R Karagas, T A Stukel, J Dykes, J Miglionico, M A Greene, M Carey, B Armstrong, J M Elwood, R P Gallagher, A Green, E A Holly, C S Kirkpatrick, T Mack, A Østerlind, S Rosso, A J Swerdlow

**Affiliations:** Section of Biostatistics and Epidemiology, Department of Community and Family Medicine Dartmouth Medical School, 7927 Rubin 462M-3, One Medical Center Drive, Lebanon, New Hampshire, NH 03756-0001, USA; Cancer Research and Registers, New South Wales Cancer Council, PO Box 572, Kings Cross 1340, Australia; National Cancer Control Initiative, 1 Rathdowne St., Carlton Vic 3053, Australia; Section of Epidemiology, Cancer Control Agency of British Columbia, 600 W. 10th Avenue, Vancouver, BC V52 4E6, Canada; Queensland Institute of Medical Research, PO Royal Brisbane Hospital, Brisbane Q4029, Australia; Cancer Epidemiology Studies, 3333 California Street, Suite 280 UCSF Box 1228, San Francisco CA, 94143-1228 USA; Good Samaritan Hospital, 407 14th Avenue, SE PO Box 1247, Puyallup WA, 98371-0192 USA; Norris Comprehensive Cancer Center, University of Southern California, 1441 Eastlake Ave., MS 44, PO Box 33800, Los Angeles, CA, 90033-0800 USA; Dermatology Clinic, Slotsgade 14, DK 3400, Hillerød, Denmark; Registro Tumori Piemonte, Via San Francesco Da Paola, 31,10123 Torino, Italy; Institute of Cancer Research, Royal Cancer Hospital, Section of Epidemiology, D Block, 15 Cotswold Rd, Sutton, Surrey SM2 5NG, UK

**Keywords:** melanoma, oral contraceptives, pooled analysis, case–control studies

## Abstract

Data regarding the effects of oral contraceptive use on women's risk of melanoma have been difficult to resolve. We undertook a pooled analysis of all case–control studies of melanoma in women completed as of July 1994 for which electronic data were available on oral contraceptive use along with other melanoma risk factors such as hair colour, sun sensitivity, family history of melanoma and sun exposure. Using the original data from each investigation (a total of 2391 cases and 3199 controls), we combined the study-specific odds ratios and standard errors to obtain a pooled estimate that incorporates inter-study heterogeneity. Overall, we observed no excess risk associated with oral contraceptive use for 1 year or longer compared to never use or use for less than 1 year (pooled odds ratio (pOR)=0.86; 95% CI=0.74–1.01), and there was no evidence of heterogeneity between studies. We found no relation between melanoma incidence and duration of oral contraceptive use, age began, year of use, years since first use or last use, or specifically current oral contraceptive use. In aggregate, our findings do not suggest a major role of oral contraceptive use on women's risk of melanoma.

*British Journal of Cancer* (2002) **86**, 1085–1092. DOI: 10.1038/sj/bjc/6600196 www.bjcancer.com

© 2002 Cancer Research UK

## 

Until about age 45 years incidence rates of melanoma in women exceed those in men, after which rates markedly rise in men but level off slightly in women (Armstrong and English, 1996). Additionally, women with a history of melanoma are at greater risk of breast cancer and vice versa (Schoenberg and Christine, 1980). These descriptive findings raise the possibility that female sex steroids may be involved in the aetiology of melanoma in women. Several epidemiologic studies have specifically addressed the possible role of oral contraceptive use in the occurrence of melanoma, but with conflicting results. Over 20 years ago, results from three cohort studies suggested a higher incidence of melanoma among women who had used oral contraceptives compared to women who had never used them (Beral *et al*, 1977; Kay, 1981; Ramcharan *et al*, 1981). Relative risk estimates ranged from 1.4 to 3.5. However, these findings were based on a relatively small number of cases, and another cohort study found no excess risk (Adam *et al*, 1981). Subsequent case–control and cohort studies (Bain *et al*, 1982; Holly *et al*, 1983, 1995; Holman *et al*, 1984; Helmrich *et al*, 1984; Beral *et al*, 1984; Gallagher *et al*, 1985; Green and Bain, 1985; Østerlind *et al*, 1988; Zanetti *et al*, 1990; Hannaford *et al*, 1991; Le *et al*, 1992; Palmer *et al*, 1992; Westerdahl *et al*, 1996; Gefeller *et al*, 1998; Smith *et al*, 1998; Feskanich *et al*, 1999) provided little evidence of an overall excess risk among ever users compared to never users, but a possible increase among long-term users (summarised in (Prentice and Thomas, 1987)). More recently, data from the Nurse's Health Studies indicated a risk of melanoma related to duration of oral contraceptive use, but only among current users (Feskanich *et al*, 1999). Use of different exposure categories makes summarising the published literature problematic, particularly for duration of use. Moreover, a number of studies collected information on oral contraceptive use but never reported the results. To clarify whether use of oral contraceptive relates to melanoma risk in women, we undertook a collaborative, pooled analysis using the original data of completed epidemiologic studies, including those for which findings on oral contraceptive use had not been published

## MATERIALS AND METHODS

### Selection of studies for analysis

We identified epidemiologic studies of melanoma completed as of July 1, 1994. In addition to an extensive literature review, we contacted an established consortium of melanoma investigators (Bliss *et al*, 1995; Ford *et al*, 1995). One criticism of the early studies was that they lacked information on potentially confounding factors. Therefore, we limited our analyses to studies that ascertained data on major melanoma risk factors including pigmentary characteristics and sunlight exposure. We further restricted our analysis to studies that involved a personal interview because questions designed for postal surveys might be phrased differently or be less complex. We also excluded studies limited to hospitalised cases since these cases might be biased because of over-representation of advanced lesions. Finally, we analysed only studies that included at least 100 women cases and 100 women controls as smaller studies would require a similar analytic effort, but contribute little to the overall analysis.

Eleven case–control studies met the analysis criteria (Beral *et al*, 1984; Holman *et al*, 1984; Gallagher *et al*, 1985; Green and Bain, 1985; Østerlind *et al*, 1988; Swerdlow *et al*, 1986; Elwood *et al*, 1990; Zanetti *et al*, 1990; Kirkpatrick *et al*, 1994; Holly *et al*, 1995; Langholz *et al*, 2000) and data were available for all but one of these (Beral *et al*, 1984). The investigators from all of the remaining studies agreed to take part. Six studies had been included in a prior pooled analysis of pigmentary characteristics and freckling (Bliss *et al*, 1995) and five in a pooled analysis of family history of melanoma (Ford *et al*, 1995). Four studies had not previously published or presented results on oral contraceptive use (Swerdlow *et al*, 1986; Elwood *et al*, 1990; Kirkpatrick *et al*, 1994; Langholz *et al*, 2000). Table 1

**Table 1:**
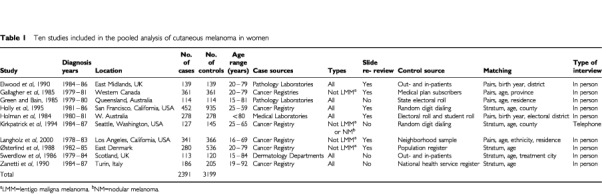
Ten studies included in the pooled analysis of cutaneous melanoma in women

provides a summary of the number of cases and controls, study locations, age ranges, study period, and method of selection of cases and controls. In each study, cases comprised histologically confirmed, incident melanoma and about half the studies verified diagnoses by a standardised histopathology review. Four studies excluded lentigo maligna melanomas; one study further excluded nodular melanomas and another was limited to superficial spreading and nodular melanomas. Only one study identified cases from dermatology clinics; all the others used central cancer registries or pathology laboratories for a specific geographic region. All studies matched on age either by pairs or by stratum. Many studies additionally matched on area of residence. Controls were sampled from population lists, random digit dialing, neighbourhood sampling, or clinic and hospitalised patients. Nine studies conducted interviews in-person and one completed interviews by telephone.

We requested a complete data set of all variables excluding personal identifiers. A copy of the original questionnaire and coding documentation accompanied each study. Materials from the Italian (Zanetti *et al*, 1990) and Danish (Østerlind *et al*, 1988) studies required English translation. Upon receipt, we transferred data sets containing information on individual study subjects onto an IBM RS600 system and converted them into SAS data files. To ensure accuracy of the received and coded data, we performed range checks and other descriptive statistics. We compared our counts with the published data and with unpublished data from previous combined analyses or those provided by the original study investigators. We reported summary statistics back to the individual study investigators for confirmation and resolution of any discrepancies.

### Study variables

We developed a common set of exposure variables by reviewing each study's questionnaire. Nine of 10 studies asked women if they ever used oral contraceptives. One study (Langholz *et al*, 2000) asked women about their contraceptive use between pregnancies. For this study, we constructed an ‘ever use’ variable by examining use in each phase of life. Two studies (Zanetti *et al*, 1990; Kirkpatrick *et al*, 1994) asked women whether they used oral contraceptives for a specified length of time, e.g., at least 6 months or for 90 days. For uniformity, we defined ‘ever use’ of oral contraceptives as 1 or more years of use and the referent category as no use or less than 1 year of use.

To assess duration of oral contraceptive use, we used the total number of years or months of use. For some studies, we needed to compute the number of months or years using dates started and stopped for each episode of oral contraceptive use. We used questions on age began and ended or year began and ended to calculate age at first use, year of first use, number of years since first use, and number of years since last use of oral contraceptives.

### Statistical methods

We used a two-stage method to analyse the pooled case–control data (Stukel *et al*, 2001). In the first stage, each study was analysed according to the original design (pair matched or frequency matched) using uniformly defined exposure variables across all studies but study-specific confounders. For pair-matched studies, we used conditional logistic regression to derive odds ratios (OR) and 95% confidence intervals. For studies that frequency matched on age, we used unconditional logistic regression and controlled for age, grouped as <35, 35–44, ⩾45 years.

The study-specific adjusted odds ratios and standard errors were combined in a second-stage linear mixed-effects model that incorporated random study effects (inter-study heterogeneity) to produce a pooled exposure odds ratio and standard error. The estimator of the pooled exposure effect is a weighted average of the individual study estimators, weighted by the inverse marginal variances; the marginal variance is the sum of the individual study-specific variances and the variance of the random study effects. To assess inter-study variability, we examined the study-specific ORs and tested for heterogeneity using a χ^2^ test. In the absence of heterogeneity, we weighted by the inverse of the study-specific variances alone since the variance of the random study effects was treated as negligible. Further details as well as comparisons of this model with a joint logistic regression are given in Stukel *et al* (2001). Additionally, we examined study factors that could contribute to heterogeneity in the odds ratios in subgroup analyses based on: (1) type of control group (e.g. restricting to population-based studies), (2) type of interview (i.e., exclusion of the Kirkpatrick *et al* (1994) study that used a telephone interview) and (3) questionnaire format (i.e., exclusion of the Langholz *et al* (2000) study that asked contraceptive history between pregnancies).

Our primary exposures of interest were ‘ever use’ of oral contraceptives of 1 year or greater duration, duration of use (<5, 5–9, ⩾10 years of use), age began (<25, 25–29, 30–34, ⩾35 years), years since first use (⩽10, 11–15, >15 years), years since last use (<2 or current use, 2–5, >5 years), and year of first use (before 1970 *vs* 1970 or after). For each, the reference category was ‘never use’ of oral contraceptives or use of less than 1 year. We also examined whether the effects of duration of use (grouped as <5 or ⩾5 years) were modified by number of years since first use (<10 or ⩾10 years). Due to small strata in this analysis, we broke the matched pairs and adjusted for age using the same categories used in the frequency matched studies. To further examine the risk of melanoma among current, long-term users, we combined the data from all studies on women less than 50 years of age. We then analysed current use, defined as use in the 2 years prior to diagnosis or interview date, among those with 10 or more years of use compared to our non-user reference group. Stratifying on ‘study’, we obtained conditional maximum likelihood estimates both for the individual studies and for all studies combined (Breslow and Day, 1980)

To the extent possible, we uniformly defined and coded multiple potentially confounding factors (summarised in Appendix 1). We used the classification scheme developed for earlier pooled analyses for eye colour, hair colour and family history of melanoma (Bliss *et al*, 1995; Ford *et al*, 1995). We grouped skin reaction to the sun into three categories (i.e., never burn, burn then tan, or always burn). Level of education was categorised into grade/high school, college, or graduate school, except in two studies (Østerlind *et al*, 1988; Kirkpatrick *et al*, 1994) that included a category for technical school. The definition and method for assessing nevi differed across studies. All but one study included a nevus count on the arms; the study done by Holly *et al* (1983) collected whole body count of nevi greater than 5 mm. For all studies, we grouped number of nevi as none, 1–4, 5–9, and ⩾10 nevi. Questions relating to sun exposure history considerably varied across studies. Therefore, we included the UV light-related factors most strongly related to melanoma risk within each study. These included history of sunburns, sun exposure, and migration to Australia (see Appendix 1).

To assess the impact of potentially confounding factors, we examined the per cent change in the age-adjusted ORs with the addition of the factor for each individual study separately as well as on the combined, pooled estimates. Using our two-staged approach, by first analysing each study separately, we were able to assess the potentially confounding effects of variables that were not included in all studies or that were measured differently (e.g., sun exposure) (Stukel *et al*, 2001). For example, to assess the impact of adjustment for nevi, we adjusted for both age and nevi in the eight studies that collected nevi information, and age alone in studies without nevi data. We did not find that addition of any potential confounder altered the pooled estimates by more 10%. Therefore, we adjusted our final estimates for only age.

Our analysis of oral contraceptives was conducted for all melanomas combined and for superficial spreading melanoma (SSM) specifically. There were too few cases of nodular melanomas or lentigo maligna melanomas to perform detailed analyses of these histologic types.

## RESULTS

A total of 2391 cases and 3199 controls were included in the analysis for the 10 case–control studies (Table 1). The prevalence of oral contraceptive use among controls (for 1 year or greater) ranged from 15% in the study from Italy (Zanetti *et al*, 1990) to 63% in the study from Northern California (Holly *et al*, 1985) (Table 2

**Table 2:**
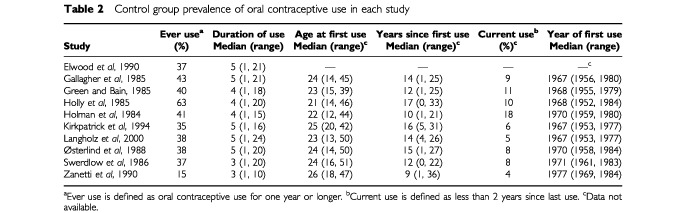
Control group prevalence of oral contraceptive use in each study

). Among controls who used oral contraceptives for at least 1 year, the median duration of use ranged from 3 to 5 years, median age at first use, 21 to 26 years, and median years since first use, 9 to 17 years (Table 2). The median year of first use among controls ranged from 1967 to 1977 across studies (Table 2).

**Figure 1:**
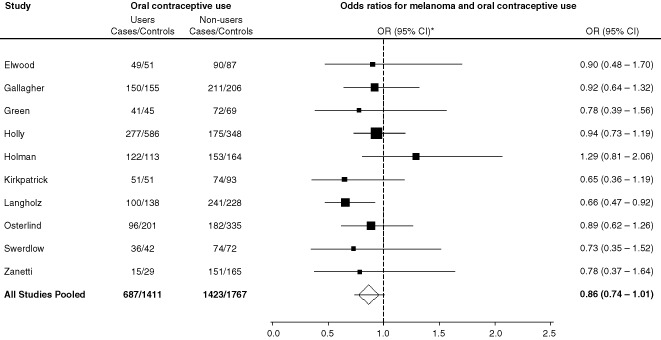
Study specific and pooled odds ratios (95% confidence intervals) for melanoma in women in relation to use of oral contraceptives. User of oral contraceptives defined as 1 or more years of oral contraceptive use and non-user as never use or less than one year of use. The individual study odds ratios are shown as a solid box and the pooled estimate as an open diamond. The size of the box is inversely proportional to the standard deviation for the odds ratio. Lines show the 95% confidence intervals.

The overall pooled odds ratio for ‘ever use’ of oral contraceptive and melanoma was close to unity (pooled odds ratio (pOR)=0.86; 95% CI=0.74–1.01) (Figure 1

). The ORs for ever use of oral contraceptives were not significantly elevated in any study, and for many studies were less than one. There was no statistically significant heterogeneity across studies (χ^2^=7.15, *P*=0.62). The findings generally were similar for superficial spreading melanoma (pOR=0.93, 95% CI=0.79–1.10, with no statistically significant heterogeneity (χ^2^=11.82, *P*=0.22). The risk of melanoma was unrelated to duration of use, age began, time since first and last use of oral contraceptives, or year of first use (Table 3

**Table 3:**
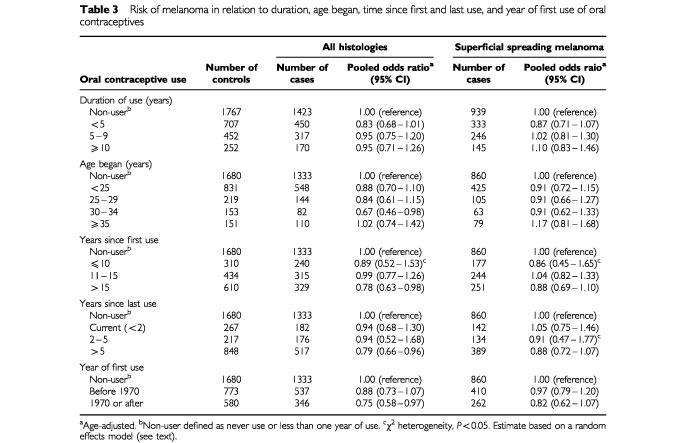
Risk of melanoma in relation to duration, age began, time since first and last use, and year of first use of oral contraceptives

). Again, we found comparable results when we restricted the analysis to SSM (Table 3). We detected evidence of statistically significant inter-study heterogeneity in only two strata : ⩽10 years since first use, and 2–5 years since last use (Table 3), and for these estimates used the random effects model. As mentioned, adjustment for potentially confounding factors (e.g., eye colour, hair colour, level of education, family history of melanoma, number of nevi, skin type and sun exposure history) did not change any of the pORs by more than 10%.

When we evaluated the effects of duration of use stratified by time since first use, we found that the pORs, for the most part, approached unity (Table 4

**Table 4:**
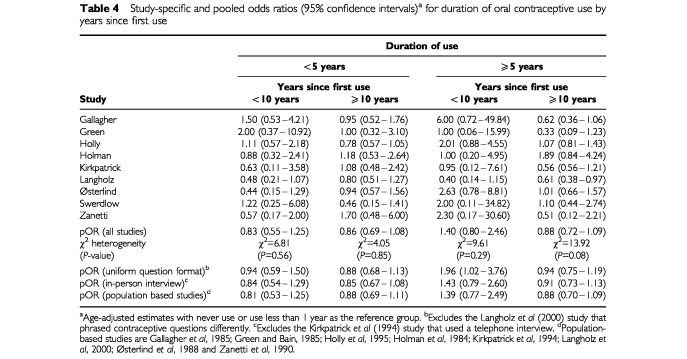
Study-specific and pooled odds ratios (95% confidence intervals)^a^ for duration of oral contraceptive use by years since first use

). However, in five studies, the ORs were elevated among women who used oral contraceptives for 5 or more years and who began using them within the past 10 years. In three studies the ORs for these women were close to one and in one study the OR was below one (Table 4). The overall pOR was 1.40 (95% CI=0.80–2.46). Excluding one study that used a differently formatted questionnaire (Langholz *et al*, 2000), the pOR for this stratum was 1.96 (95% CI=1.02–3.76). While the individual study ORs appeared to vary, there was no statistical heterogeneity in the ORs across studies (χ^2^=9.61, *P*=0.29). Again, none of these odds ratios changed by more than 10% with the additional potentially confounding factors. Results were similar for SSM (data not shown).

To examine the effects of long-term oral contraceptive use among current oral contraceptive users, we pooled data on women younger than age 50 years. In total, there were 61 cases and 87 controls who were current oral contraceptive users with 10 or more years of use. Combining data into a single conditional logistic model and adjusting for ‘study’, the odds ratio among current, long-term users compared to non-users was 0.91 (95% CI=0.62–1.32). There was no evidence of inter-study heterogeneity (*P* value for heterogeneity=0.82).

## DISCUSSION

In our combined re-analysis of 10 case–control studies of melanoma in women, we found no overall association between use of oral contraceptives and melanoma risk. Studies included in our analysis represent the largest case–control studies of women completed by 1994 that personally interviewed women about their use of oral contraceptives along with other melanoma risk factors. By applying strict selection criteria, we attempted to minimise inter-study heterogeneity in results that could arise from design characteristics. Still, some aspects varied between studies, such as method of case or control selection (i.e., clinic *vs* population-based), type of interview (in-person *vs* telephone) and questionnaire format (i.e., simple to complex). However, we observed negligible difference in our findings according to these factors.

There are reasons for suspecting that sex-steroids might affect women's risk of melanoma. Melanomas rarely occur before the age of 15 years, but incidence rises with age thereafter (Armstrong and English, 1996). Also, interestingly, rates are higher in women than men prior to age 45 years, and after age 50 years, the rate of increase with age slows in women but rises steeply in men (Armstrong and English, 1996). The shape of the age incidence curve for melanoma resembles that of breast cancer and suggests a possible role of sex steroids and reproductive factors in the pathogenesis of both of these tumours. Indeed, women with a history of breast cancer appear to be at increased risk of melanoma and vice versa (Schoenberg and Christine, 1980). Increased pigmentation, melanocytic proliferation, and tumour growth have been observed following oestrogen (and to a lesser extent progesterone) administration in experimental laboratory animals (Snell and Bischitz, 1960; Lopez *et al*, 1978). Another intriguing finding is that tamoxifen, a selective oestrogen receptor modulator used in the treatment of breast cancer, improved the median survival time of patients with metastatic melanoma when used with dacarbazine – an effect largely observed in women (Cocconi *et al*, 1992). Oestrogen-binding receptors have been detected in melanomas and benign nevi of melanoma patients; although they do not appear to be ‘true’ oestrogen receptors (Walker, 1990).

There have been positive findings in some studies conducted after we began our collaborative investigation, and hence could not be included in the present analyses. In the Nurses Health Study (NHS) and NHS II cohorts that involved postal surveys, an elevated risk of melanoma was found in relation to current but not past use of oral contraceptives (Feskanich *et al*, 1999). Compared with never users, a relative risk of 3.4 (95% CI=1.7–7.0) was observed among current oral contraceptive users who had used them for 10 years or more. However, even in these large cohorts, the numbers of exposed and unexposed cases were relatively small (six exposed cases in NHS and eight in NHS II). Two other recent and relatively large case–control studies reported from Connecticut and Sweden (Westerdahl *et al*, 1996; Smith *et al*, 1998) observed no relation between ever use or duration of oral contraceptive use and risk of melanoma. But, neither of these studies specifically examined current *vs* past use of oral contraceptives. Another case–control study restricted to women less than 55 years of age found an elevated risk (OR=1.5; 95% CI=1.0–2.1) in relation to 5 or more years of oral contraceptive use, among those who began use 10 or more years before (Beral *et al*, 1984). While this study met the eligibility criteria for our analysis, the original data were no longer available. It seems unlikely that the addition of the 287 cases and 574 controls from this study, would have dramatically altered our findings based on a total of 2391 cases and 3199 controls.

Several earlier studies did not collect data on potentially confounding factors such as sun exposure history or pigmentary characteristics or nevi and thus were excluded from our analysis (Beral *et al*, 1977; Adam *et al*, 1981; Kay, 1981; Ramcharan *et al*, 1981; Bain *et al*, 1982; Holly *et al*, 1983; Helmrich *et al*, 1984; Hannaford *et al*, 1991; Palmer *et al*, 1992). The results of these studies vary. In two studies, an increased risk of melanoma was observed among long-term users who began using oral contraceptives 10 or more years ago (Bain *et al*, 1982; Beral *et al*, 1984). In a hospital-based study, Palmer *et al* (1992) found a modestly elevated risk among current oral contraceptive users that was not statistically significant. In our data, the one possibly raised risk was among the subgroup of long-term users, who started taking oral contraceptive within 10 years of diagnosis. While these findings appear consistent with the NHS results (Feskanich *et al*, 1999), we found no evidence of an increase risk associated with current oral contraceptive use (i.e., use within the past 2 years) even among the subgroup of long-term, current users.

Increased detection among women who used oral contraceptives is a plausible explanation for any observed association between melanoma and oral contraceptive use. In our analysis, we found little effect on our risk estimates when we adjusted for sociodemographic factors such as level of education that could be related to screening behaviour. Indeed, we extensively evaluated the impact of multiple factors on the odds ratios, but none appreciably altered the results. In conclusion, our pooled epidemiologic data do not suggest a major role of oral contraceptives on women's risk of melanoma.

## Figures and Tables

**Appendix 1:**
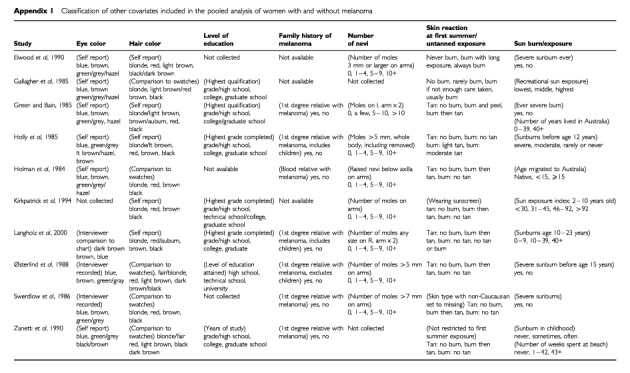
Classification of other covariates included in the pooled analysis of women with and without melanoma.

## Appendix

Appendix 1
